# Misapplied Sympathy

**Published:** 1878-10

**Authors:** 


### MISAPPLIED SYMPATHY.

"He's a smart fellow; what a pity he drinks!"

A stereotyped expression that can be improved upon and at the same time made truthful:

He's an idiot, therefore, *natural* that he should drink.

We waste a deal of sympathy, time and money, upon whiskey guzzlers; treasures that were better applied to worthier objects.

We can name at least a half dozen young men in this city who are supported, caressed, and semi-lionized by well-meaning people who try to think they are reforming or reclaiming them. Now and then, a genuine success is obtained in the reformation, returning to society a useful man. The instances are so rare, however, that the reforming business is scarcely worth the following. How many men do you know of, once genuine drunkards, that are now wholly reformed and exemplary members of society? How many? Six? Scarely. Four? Improbable. One? Barely possible.

Gough, you may point to as an illustrious example. Aye, but Gough had brains.

One more case, [occurring in our own city] and we have the sum total of genuine recoveries from habitual drunkenness, out of a list of hundreds that have come under our observation.

The indiviual last referred to, reformed and will so remain, simply because he has brains—brains to spare, brains to carry on a business involving millions of money. But when you attempt to reform a poor, miserable sot with but a thimbleful of brains, that meagre quantity becomes so thoroughly pickled in rum, by a very few debauches, that the owner ever afterward, is utterly unable to frame an idea above a desire for a glass of grog.

We are of the opinion that many young men are inveigled into periodical intoxication by the prodigal sympathy of sober friends.

The young man may have cultivated a talent for music, art, or some one of the sciences, in any or all of which he is not a whit more skilled than some other young man of steady habits; yet, because he is now and then found drunk, many willing hands are outstretched to give him profitable employment, and boost him forward toward success. Naturally enough, he fails. He lacks brains; [else would he not have been found drunk] therefore, the only thing left him to do is to get drunk again, and rely upon his friends for another caress—a new suit of clothes and a fresh start, while all look on admiringly and exclaim—

"So smart, what a pity he drinks!"

Bah! We are sick of such nonsense, and prefer to have charitably inclined people turn their attention to more worthy objects. Aid young men who are smart, and never drink. Those are the ones who will give you returns for well bestowed help that will be a lasting pleasure to you. Do not set a premium upon drunkenness longer. Do not let the worthy young man see that he must debauch himself before his abilities are appreciated. To praise the accomplishments of the former, or to aid him with your purse, if he requires it, is worth more than all the sympathy and assistance wasted upon one of those smart but inebriated dolts.

